# Percutaneous endoscopic gastrostomy insertion in patients with neuromuscular disease-a single centre experience of a novel technique

**DOI:** 10.3389/fgstr.2026.1814988

**Published:** 2026-06-29

**Authors:** Anissa Faher, Melanie Gunn, Timothy L. Williams, Christian Dipper, Lindsay McGowan, Catherine Rimmer, Ben Messer

**Affiliations:** Newcastle upon Tyne Hospitals NHS Foundation Trust, Newcastle upon Tyne, United Kingdom

**Keywords:** endoscopy, neuromuscular disease (NMD), non-invasive ventilation (NIV), nutritional support, PEG

## Abstract

**Background:**

Neuromuscular diseases (NMD) are often complicated by bulbar dysfunction limiting nutritional intake, and ventilatory failure requiring non-invasive ventilation (NIV). Although nutritional difficulties can be overcome with gastrostomy insertion, this has its own challenges since percutaneous endoscopic gastrostomy (PEG) requires endoscope access via the oral cavity with air leak-associated risk of hypoventilation and respiratory complications in patients with NIV-dependent NMD. Developing a mechanism for PEGs under sedation with concurrent NIV use has remained a clinical challenge.

**Aim:**

We report a change in practice in our hospital with the introduction of the endoscopy elbow (EE), an adjunct device enabling simultaneous use of NIV during PEG with minimal air leak.

**Methods:**

This retrospective service evaluation analysed data of PEGs in NIV-dependent NMD in a tertiary referral hospital over a ten-year period measuring respiratory complications, critical care admissions, time from referral to procedure, hospital stay, and survival times.

**Results:**

The EE enabled a successful transition from PEGs conducted under general anaesthesia to PEGs with sedation and NIV in the endoscopy suite, with statistically significant reduction in respiratory complications and critical care admissions for all patients with NMD and improved six-month survival in patients with motor neurone disease. There was no improvement in length of time from referral to procedure, nor hospital stay.

**Conclusions:**

The EE provides a novel technique with improved outcomes for respiratory complications and critical care admissions. This technique may be applicable to other centres caring for similar patient cohorts, although local protocols and resources should be considered.

## Introduction

Progression of neuromuscular diseases (NMD), such as Motor Neurone Disease (MND) and congenital myopathies, can lead to the development of bulbar dysfunction requiring assistance with feeding to meet nutritional requirements (1). Gastrostomy enables the insertion of a feeding device through the skin of the abdomen into the stomach and can aid in overcoming the challenge of aspiration risk as well as supporting nutrition. There are several methods of gastrostomy insertion such as percutaneous endoscopic gastrostomy (PEG), radiologically inserted gastrostomy (RIG) and surgically assisted gastrostomy (2). The method chosen often depends on patient anatomy as well as centre-dependent established practices. In general, a PEG procedure requires the administration of sedative or general anaesthesia (GA) medications which have respiratory depressant effects and can result in respiratory complications (3, 4).

Patients with NMD can develop ventilatory failure, which can be treated with non-invasive ventilation (NIV), an intervention shown to increase survival in MND and Duchenne muscular dystrophy (DMD) and quality of life in MND (5, 6). Patients who have developed ventilatory failure are at increased risk of hypoventilation following the administration of GA and sedative medications. The challenge of gastrostomy in patients receiving NIV during the procedure arises from the need for simultaneous access to the upper airway for the endoscope and the NIV mask (7, 8). Gastrostomy insertions under GA have been safely reported in this subgroup but not without significant risks and additional concerns about disease progression in MND (9). An approach of no sedation could be taken but this is uncomfortable for the patient especially if the procedure is prolonged. Finally, the procedure could be undertaken with sedation but without NIV though this may result in significant respiratory depression and could not be undertaken in patients dependent on NIV.

The best practice for PEG has yet to be established and various groups have reported safe and effective methods of PEG in patients with NMD (7, 8, 10). A GA offers the advantage of patient comfort but has significant risks of respiratory morbidity post-operatively (11). Improved survival has been reported with non-invasive positive pressure ventilation under conscious sedation (12). There are therefore important reasons to try to undertake PEG with sedation and NIV *in situ*.

This study reports a change in practice in a tertiary referral hospital with the introduction of an adjunct device into the NIV circuit which enables the simultaneous use of NIV, with minimal air leak, during PEG. We have used the term ‘Endoscopy Elbow’ (EE) to describe this and it is an existing device primarily used to facilitate bronchoscopy.

## Methods

This is a retrospective cohort study of patients with NMD who underwent PEG in a tertiary referral hospital from 2014-2024. Patients were using NIV or had been referred to the local home mechanical ventilation service with respiratory insufficiency for consideration of NIV. Data were extracted from the hospital electronic patient record (EPR). Data collected included time from referral to PEG, length of hospital stay, critical care admission, frequency of respiratory complications and six-month survival. All data collected were from the Newcastle upon Tyne Hospitals NHS Foundation Trust, United Kingdom.

**Figure 1:**
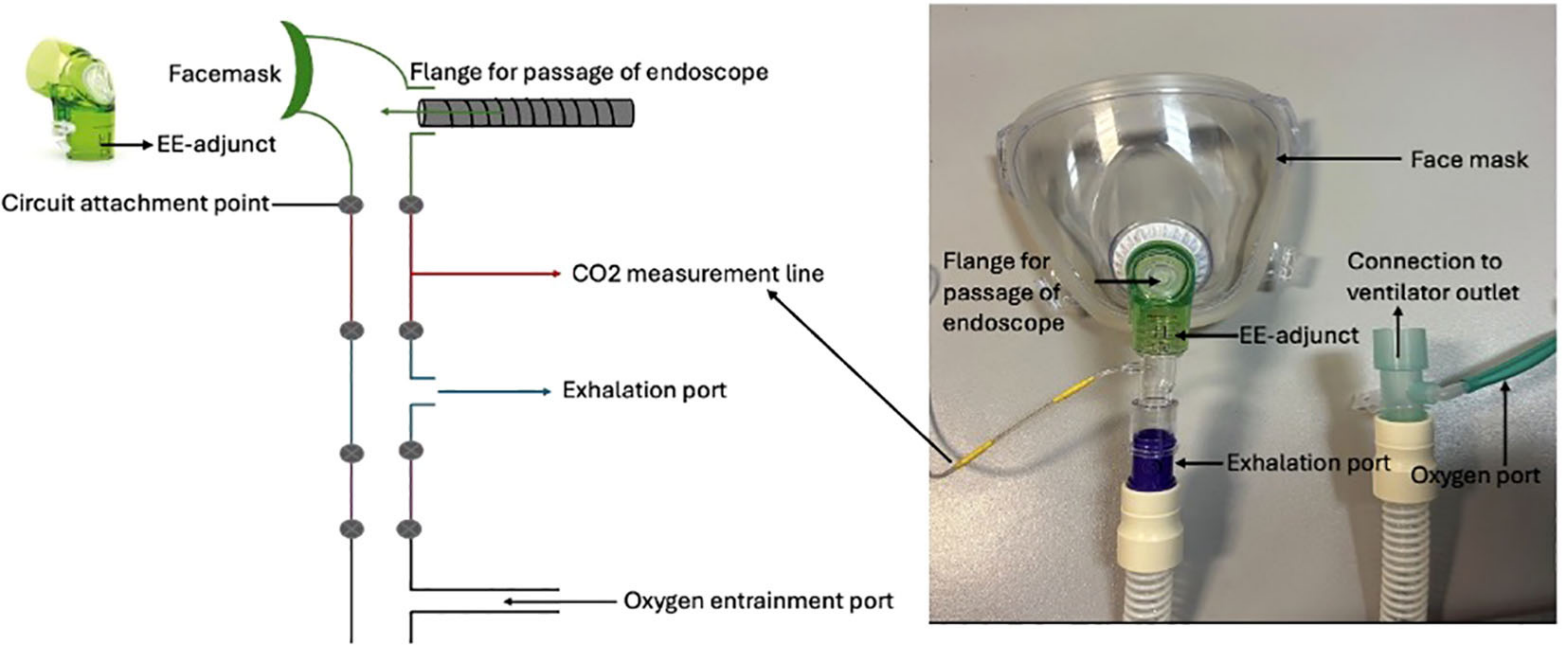
Visual representation of the EE circuit with endoscope passage. The endoscopy elbow used is also shown.

The commercial name of the endoscopy elbow is ‘Bronchoscopy Elbow for the PerfoMax mask’ and was sourced from Philips Healthcare, Netherlands, and were incorporated into the NIV/endoscopy circuit as outlined in **Figure 1**.

Practice evolved over time, and data is divided into three methods to represent the change in PEG practice based on technique.

- Method 1 involved PEG with the use of a variety of non-EE assisted ventilation techniques including pre-existing tracheostomy, NIV using a nasal mask, PEG with high flow nasal oxygen (HFNO) and RIG. Patients were monitored according to Association of Anaesthetists of Great Britain and Ireland guidance (AAGBI) (13). Medication involved a propofol target-controlled infusion (TCI).
- Method 2 involved the use of an EE assisted NIV with PEG under sedation in the operating theatre. Patients were monitored with oxygen saturation monitoring and end tidal carbon dioxide (ETCO_2_) monitoring. Medication was with a single bolus of propofol and glycopyrronium.
- Method 3 involved the use of an EE assisted ventilation with PEG under sedation in endoscopy. Patients were monitored with oxygen saturation monitoring and end tidal carbon dioxide monitoring. Medication was with a single bolus of propofol and glycopyrronium.

A summary of the above methods is shown in **Table 1**:

**Table 1:** A comparative table of methods 1, 2 and 3.

	Method 1	Method 2	Method 3
Ventilation strategy	Variable (pre-existing tracheostomy, nasal mask, high flow nasal oxygen )	NIV	NIV
Procedural setting	Theatre	Theatre	Endoscopy
Anaesthesia/ sedation approach	GA with propofol target-controlled infusion (TCI)	Sedation with single bolus of propofol and glycopyrronium	Sedation with single bolus of propofol and glycopyrronium
Monitoring	AAGBI guidance	Oxygen saturations, ETCO_2_	Oxygen saturations, ETCO_2_

**Figure 2:**
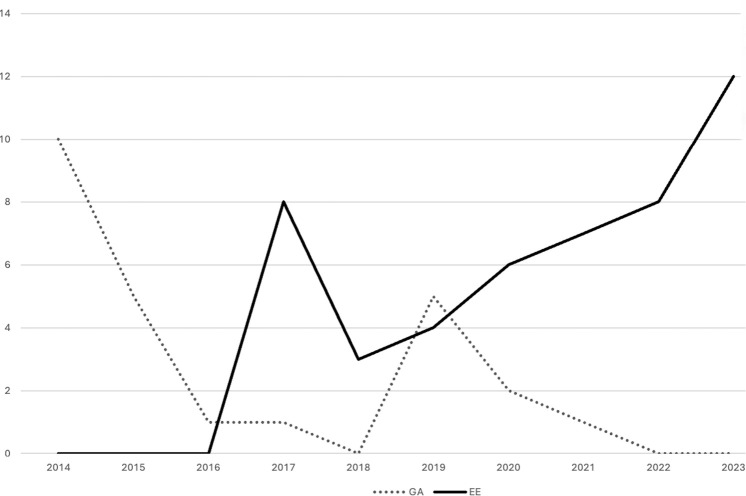
Change in practice over time from GA to EE.

These three methods do not precisely coincide with distinct time points as PEG placement under GA was necessary in later years mainly due to anatomical requirement or procedures performed in patient with learning disability. This is demonstrated in **Figure 2** in the results section.

Fishers exact test was used for statistical analysis of the data, using Microsoft (Redmond, Washington, USA) Excel 365. The Kaplan Meier curve was used to analyse six-month survival of MND patients in the cohort and plotted using Prism Graphpad.

## Results

Data from 91 patients with NMD were analysed. Demographic data are presented in **Table 2**.

**Table 2:** Diagnosis, median age, age range and gender demographics of patient cohort and total number of patient and participants in each method.

	MND N= 56	DMD N=14	Myopathy N=5	SMA N=4	Other N=12
Age in years Median (Age Range)	64 (34-80)	23 (17-41)	50 (27-68)	24 (18-33)	60 (22-75)
Female: Male	1:1.3	0:1	1:4	1:3	1:1.4
Method 1 (N = 42)	21	10	3	3	5
Method 2 (N = 24)	17	0	2	1	4
Method 3 (N = 25)	18	4	0	0	3

MND, motor neurone disease; DMD, Duchenne muscular dystrophy; Myopathy, myofibrilla, nemaline, bethlam myopathies and hereditary myopathy with early respiratory failure; SMA, spinal muscular atrophy. Other: cerebral palsy, facioscapulohumeral muscular dystrophy, multiple system atrophy, becker muscular dystrophy, spinal cord injury and cerebrovascular accident.

All PEGs for NIV-dependent NMD patients were inserted under GA in the operating theatre in 2014. As shown in **Figure 2**, a decline in GA use is noted over time, with a concurrent rise in EE from its introduction in 2016. By 2023, all PEGs were performed with the use of EE-assisted ventilation. Spikes in GA use over the years have usually been due to ventilation-independent reasons including the anatomical requirement for laparoscopically-assisted PEGs or PEG in patients with a learning disability.

Due to the retrospective nature of this study and limitations of electronic system, accurate and consistent drug doses were not retrievable. However, during methods 2 and 3, median doses of propofol and glycopyrronium were 50 mg and 200 mcg respectively. During method 1, a propofol TCI was used with an initial target of 2 micrograms/mL.

Similarly, consistent accurate physiological data were not collected. Frequent air leak from the open mouth was observed, accompanied by desaturation in patients for whom a nasal mask was used during method 1. No significant air leak or desaturation was seen during method 2 and 3 when oxygen requirements were between 0–2 l/minute, entrained into the NIV circuit.

The study results are summarised in **Table 3**:

**Table 3:** Summary of outcomes measured in this study under each different method.

	Method 1		Method 2		Method 3
Critical Care Admissions n (%)	33(82.5)		13 (54.2)		3(12)
Respiratory complications n (%)	9 (22.5)		0 (0)		3 (12)
Length of hospital Stay (days)	6 (1-129)		7 (2-64)		7 (2-41)
MND 6-month survival (%)	40.0%		65.0		72.0
Comparison		Outcome		OR (95%CI), p-value	
Method 3 vs Method 1		Critical care admissions		0.03 (0.006-0.14) p <0.05	
Method 3 vs Method 2		Critical care admissions		0.1 (0.03-0.49) p<0.05	
Method 3 vs Method 1		Respiratory complications		0.2 (0.06-0.89) 0.03	

Categorical variables are presented as number (percentage). There were some missing data from the electronic patient records. This was accounted for in the statistical analysis.

Due to non-normal distributions for length of hospital stay, these are presented as median (range).

82.5% of patients who underwent PEG in method 1 required planned admission to critical care compared to 54.2% in method 2 and 12.0% in method 3. There was a significant reduction in planned critical care admission in method 3 compared to method 1, Odds Ratio 0.03(95%CI 0.006-0.14) p <0.05, Fisher’s exact test. A significant reduction in critical care admissions was also seen in method 3 compared to method 2, Odds Ratio 0.1(95%CI 0.03-0.49) p<0.05, Fisher’s exact test.

22.5% of patients who had PEGs with a non-EE assisted ventilation experienced respiratory complications including aspiration pneumonia, lobar collapse, respiratory failure, pneumothorax and respiratory infections leading to critical care admission as well as death during the procedural admission. Only 6.1% of patients who had PEGs with EE-assisted ventilation experienced a respiratory complication, with the only complication being hospital acquired pneumonias (HAP) Odds Ratio 0.2 (95%CI 0.06-0.89) p= 0.03, Fisher’s exact test.

The use of EE showed no difference in length of time from referral to PEG insertion, with median waiting time of 32 days in method 1, 45 days in method 2 and 42 days in method 3. Median length of hospital stay was not significantly different between methods (6 days in method 1, 7 days in method 2 and 7 days in method 3).

**Figure 3:**
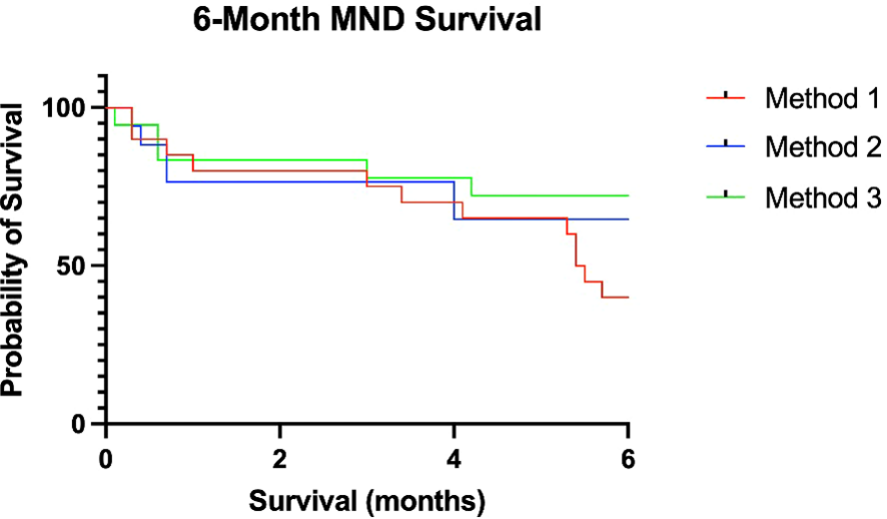
MND 6-month survival Kaplan-Meier curve. Method 1, non-EE-assisted ventilation under Method 2, EE -assisted ventilation in operating theatre. Method 3, EE-assisted ventilation in endoscopy suite.

Patients with different NMDs have varying life expectancies. The majority of patients (61.5%) in this study had MND. Six-month survival for patients with MND was 40% in method 1, 65% in method 2 and 72% in method 3 as shown in **Figure 3**.

## Discussion

In this review of practice, we report a change in practice at a large tertiary referral hospital over time from predominantly GA-based PEGs conducted in the operating theatre to the use of sedation with NIV and an EE adjunct conducted in the endoscopy suite. We report a reduction in critical care utilisation and in post-operative respiratory complications during this change in practice, with the use of EE-associated ventilation during PEG. These improved outcomes may be related to avoidance of GA and associated respiratory and disease-specific complications.

No significant improvement in the time between referral and PEG procedure was demonstrated. Similarly, no significant improvement in hospital length of stay was demonstrated. This is possibly due to both these timings being dependent on factors which the PEG technique could not plausibly be expected to influence. These include the urgency of the procedure, staff and bed availability and delays caused by training and the provision of adequate care packages following PEG insertion.

The majority of patients undergoing PEG in this tertiary referral hospital had MND. In that context, the PEG technique itself is unlikely to affect long-term survival. In the short term, six-month survival rates for MND patients were higher with the use of EE-assisted ventilation during PEG in this study. We chose six-month survival in MND patients alone because the proportion of MND patients was different between the methods used (**Table 2**). Comparisons across diagnostic groups, where survival times are significantly different, is unlikely to be useful or valid. Six-month mortality was chosen because these MND patients had respiratory insufficiency and median survival after commencing of NIV in MND is in the order of seven months (5, 14), longer term survival could not plausibly be influenced by PEG insertion method. Although difficult to establish the mechanism by which our novel technique may have impacted 6-month survival in patients with MND, the impact of GA on MND progression is incompletely understood (9); and therefore, these results were included as an explorative outcome.

The change in our method of PEG placement over time had several drivers. We moved away from GA due to concerns about safety and disease progression (4, 9). Anecdotally, we noted significant mouth leak and desaturation during use of a nasal mask to deliver NIV during PEG placement. We chose PEGs as they are bumper retained and therefore have a lower risk of post-procedural complications such as displacements, compared to ballon retained RIGs (1% risk of displacement in bumper retained gastrostomy compared to 31% risk of displacement in RIGs) (15). We also observed one case of colonic perforation during RIG placement. Although a case series has found trans nasal endoscopy facilitated gastrostomy safe, a third of the patients in the series did not have a pathology which would require NIV and the study did not comment on the number of patients receiving NIV during the procedure (16).Finally, the change from PEG placement in the operating department to the endoscopy department was largely driven by an improved availability in endoscopy compared to theatre.

We acknowledge several limitations of this review of practice. The retrospective nature of this study introduces the potential of information bias, as the documentation of the key data varied between clinicians. To overcome this, structured fields were used when available and free text notes such as ward round entries, operation notes and clinic notes were used where necessary. Additionally, confounding variables such as disease severity and patient demographics were not adjusted for, thus the results may partly reflect underlying patient characteristics. Furthermore, the EPR did not allow for accurate collection of physiological data. Anecdotally, peri-procedural physiological instability was more common during GA procedures and in procedures undertaken with a nasal NIV mask compared to those conducted with NIV using the EE. However, improved data collection in the future may demonstrate this definitively. Similarly, it was not possible to collect accurate drug doses especially for the target-controlled infusion concentrations. It was, therefore, only possible to report median rather than mean drug doses when given as a bolus.

Although planned critical care admissions were not protocolised, there is UK national guidance that patients with DMD should be cared for following a GA or procedural sedation in a critical care environment (17). This guidance was routinely followed for all patients after the predominantly GA technique associated with method 1. However, we recognised that the physiological stability associated with the use of the EE meant that patients could be safely managed outside of a critical care environment following PEG placement.

Furthermore, given the retrospective nature of our study, it is possible that during the timescale of the study, improvements in clinical care and practice may have occurred which could contribute to the positive outcomes observed.

An economic analysis was not undertaken, although theatre and critical care utilisation decreased over time suggesting that a cost benefit with a move to EE assisted NIV is likely.

Finally, given the above limitations of our retrospective cohort study, a multivariate statistical analysis was not undertaken.

## Conclusion

The method of sedation and respiratory support used during PEG has changed over time in this tertiary referral hospital study. The use of NIV with an EE adjunct is a novel approach, appears safe and results in reduced critical care utilisation and respiratory complications. Given the improved outcomes with the use of the EE adjunct, this technique may be applicable to other centres caring for similar patient cohorts, although local protocols and resources should be considered.

## Data Availability

The datasets generated and analysed during the current study are available from the corresponding author on reasonable request, subject to institutional approval, data governance regulations, patient confidentiality requirements, and review of the proposed purpose and method of data use. Requests to access the datasets should be directed to a.faher@nhs.net.
